# Pediatric IgE mediated food allergies and ethnic group inequalities: A scoping review^☆^

**DOI:** 10.1016/j.waojou.2026.101395

**Published:** 2026-05-12

**Authors:** Bashayr H. Alzughaibi, Louise J. Michaelis, Mark S. Pearce, Andrea Fairley, Emma Priestley, Sophie D. Hadfield, Nicola Heslehurst

**Affiliations:** a Population Health Sciences Institute, Newcastle University, UK; b Great North Children's Hospital, Newcastle Upon Tyne NHS Foundation Trust, UK; c School of Biomedical, Nutritional and Sports Sciences, Newcastle University, UK; d Postgraduate Education Department, University Hospital of North Tees, UK

**Keywords:** Ethnicity, Healthcare disparities, Food hypersensitivity, Child

## Abstract

**Background:**

The prevalence of pediatric food allergy ranges between 8% and 10% depending on age, geographic location, and the diagnostic criteria. There is considerable variation in clinical manifestation, with disparities in symptoms and phenotypes related to race, ethnic group, and coexisting allergic disease(s). The current evidence on ethnic group and racial differences remains limited and inconsistent. This scoping review aimed to explore the existing evidence on ethnic group and race health inequalities in pediatric IgE mediated food allergies in high-income countries.

**Methods:**

We conducted a systematic search using MEDLINE, Embase, and CINAHL, inclusive of grey literature from database inception to November 2024. Observational and qualitative studies reporting data for children aged 0–19 years with professional diagnosis of IgE-mediated food allergy, and ethnic group or race in high-income countries, without language restrictions, were included. Screening and data extraction were performed independently in duplicate. Results were mapped thematically and reported descriptively. The review was conducted using Joanna Briggs Institute (JBI) methodology for scoping reviews and reported using the Preferred Reporting Items for Systematic Reviews and Meta-Analyses extension for Scoping Reviews (PRISMA-ScR) guidelines.

**Results:**

Searches identified 4373 results, and 37 studies were included reporting pediatric IgE mediated food allergy and ethnic group data. Only 17 studies explicitly reported ethnic group differences, with most studies focused on prevalence. Nine studies examined prevalence differences, generally showing higher rates of food allergy among Black and Asian children compared with White children. Three studies examined differences in access to care and management between Black and Hispanic/Latino children and White children. Patterns showed White children had greater access to allergen-free foods and were over-represented in oral immunotherapy and more likely to receive epinephrine. Five studies examined health outcome severity, with some evidence suggesting Black children had the highest rates of food-induced anaphylaxis, and greater odds of asthma and allergic rhinitis compared with White children.

**Conclusion:**

Evidence to-date suggests there are ethnic group and racial differences among children with IgE-mediated food allergy related to prevalence, access to care and management, and health outcomes. However, there is incomplete and inconsistent reporting of ethnicity. Only 17 of the 37 included studies that included ethnic group and race data analyzed and reported ethnic group differences. This limits the field's capacity to understand inequalities and additional care requirements for minoritized and racialized ethnic groups. Further research is vital to inform the development of allergy services for children to ensure they have equitable access to care and treatment, as well as optimal health outcomes, regardless of ethnic group and race.

## Introduction

Food allergy (FA) is defined as an immune-mediated adverse reaction to certain foods and is a potentially life-threatening condition.^1^ Immunologic reactions can be categorized into 3 groups based on the nature of the immune response: IgE-mediated, non-IgE mediated, and their combination.^2^ In recent decades, global prevalence of IgE-mediated FA has increased significantly, resulting in a substantial burden on patients, their families, and healthcare systems.^3^ Data suggest that the prevalence of FA ranges from 8% to 10% depending on age, geographic location, and the criteria used for assessing FA.^4^ However, many studies use self-reports or parent reports, making it difficult to determine the exact prevalence of FA in a population.^5^ Despite the challenges of obtaining precise prevalence estimates, prevalence of FA is increasing in certain regions such as the United States of America (USA), the United Kingdom (UK), China, and Australia.^6^^,^^7^

There is considerable geographic variation in FA epidemiology, clinical manifestations, and disparities in symptoms and phenotypes related to different races, ethnicity groups, ages, and coexisting allergic diseases.^7^ Variations in regional prevalence have enhanced our understanding of the environmental role in the etiology of FA, prompting efforts to better understand the causes and mechanisms underlying FA to improve diagnosis, and to develop prevention and treatment strategies.^7^^,^^8^ Studies from high-income countries (HICs) report differences across ethnic groups in pediatric FA prevalence, sensitization patterns, and diagnosis.^4^^,^^9^^,^^10^ There is also evidence of intersecting inequalities in FA patterns, for example, minoritized racial groups with lower socioeconomic status were reported to have lower rates of formal FA diagnoses and epinephrine prescriptions but had a higher incidence of food-induced anaphylaxis.^4^^,^^9^, ^10^, ^11^, ^12^ Evidence from the UK national registry data indicates that children from minoritized ethnicities or living in households with lower socio-economic status had longer wait times for specialist referrals and were less likely to be referred to tertiary allergy centers.^13^ The literature examining ethnic health inequalities in pediatric FA remains heterogeneous. Studies differ in their design, data sources, approaches to defining and categorizing ethnicity, and outcomes assessed.^14^ Given the evidence on differences in prevalence of IgE-mediated FA in this population, systematic exploration is needed to examine the extent of the evidence, and gaps in to inform pediatric FA service development. This scoping review aimed to explore the existing evidence on ethnic group and race health inequalities related to pediatric IgE-mediated FAs.

## Methods

A scoping review was conducted to explore the breadth of the literature, map and summarize the evidence, and inform future research and service development needs. This methodology allows for a deeper understanding of the scope and coverage of a particular topic.^15^ The review protocol was registered in the Open Science Framework (OSF) to ensure transparency and accessibility (https://doi.org/10.17605/OSF.IO/FK72N). The review was conducted in line with the Joanna Briggs Institute (JBI) methodology for scoping reviews and reported using Preferred Reporting Items for Systematic reviews and Meta-Analyses extension for Scoping Reviews (PRISMA-ScR) guidelines.^17^

“Population, Concept and Context” (PCC) was used to formulate the search strategy and inclusion criteria.^18^ The PCC components for this review are Population (P) pediatric patients aged 0–19 years; Concept (C) IgE mediated FAs and ethnic group health inequalities; and Context (C) HICs (the PCC framework is defined in Appendix 1 and the list of HICs provided in Appendix 2). HICs were selected to facilitate exploration of ethnic group inequalities in broadly comparable healthcare contexts given that healthcare systems are key social determinants of health.

### Eligibility criteria

Studies were eligible for inclusion if they reported primary data on pediatric populations with confirmed IgE-mediated FA or food-induced anaphylaxis (Appendix 3). Included studies explicitly examined ethnic group or race inequalities in FA using observational, qualitative, or mixed method designs, while review articles were excluded. Only studies with full-text availability were included, where full articles could be obtained via subscription, open access, or paid access. Abstract-only publications, such as conference abstracts, were excluded as they do not provide sufficient methodological or outcome information.

### Information sources

A three-step search strategy was used.^18^ First, an exploratory search identified 9 tracer papers that were indexed in Medline and used to refine the strategy. Second, a Medline search was developed using keywords, Medical Subject Headings (MeSH), Boolean operators, truncation, and wildcard symbols such as ethnic∗, inequalit∗, and allerg∗. The strategy was iteratively refined until all tracer papers were retrieved. The Medline strategy was adapted for Embase and CINAHL (Appendix 4). Searches were not restricted by language or publication year. Grey literature sources were searched. These included the King's College London Research Portal, Open Access Theses and Dissertations, and Google Scholar. Finally, forward and backward citation chaining of all included studies was performed to identify any relevant records not picked up by the database searches.^18^

### Selection of source of evidence

Search results were imported into EndNote 21 for deduplication and then into Rayyan for title and abstract screening.^19^ Four reviewers (BA, AF, NH, EP) independently assessed titles and abstracts using predefined inclusion criteria. The screening was conducted in duplicate, and BA screened all results for consistency. An Excel tool was developed for full-text screening tailored to the scoping review inclusion criteria and 3 reviewers (BH, AF, EP) contributed to piloting using 19 randomly selected studies. Following the pilot, minor revisions were made before full screening commenced. Five reviewers (BA, AF, NH, EP, LM) assessed full texts independently, and each article was screened in duplicate; BA screened all results for consistency. Studies that met all eligibility criteria were included; disagreements were resolved through discussion. Screening results were recorded and are presented in a PRISMA flow diagram.^20^

### Data extraction

Three reviewers (BA, EP, SH) independently extracted data using a standardized Excel form tailored to the review aims and supported by detailed guidance notes.^21^ BA extracted data for all results for consistency. The form was piloted by reviewers to ensure consistent interpretation and refined before full data extraction. Data extraction was iterative, allowing updates throughout the review.^21^

Data extraction was divided into 2 components. First, study characteristics including author, year, country, design, aim, sample demographics, socioeconomic status, family history of FA, diagnosis method, FA types, outcomes, and where ethnic groups were explicitly analyzed. Second, the reported findings, including ethnic group comparisons, prevalence by FA types, access to FA services, health outcomes, socioeconomic status analysis, and other findings were extracted. Unreported data were recorded as “N/R”, and ethnic group categories were extracted using the terminology as reported in the included studies.

### Reporting the data

A descriptive summary of identified studies on children's IgE-mediated FAs and ethnicity is presented using narrative-tabular formats.^18^ Reporting is structured in 2 stages. First, mapping is reported for studies where ethnic group data is included in the studies, but no further analysis was reported on ethnic group inequalities. Second, a narrative overview is presented to identify the available evidence and gaps on ethnic group inequalities.

Findings were organized across 3 thematic domains: (1) prevalence differences, (2) access to care and management, and (3) health outcome.

## Results

**Fig. 1:**
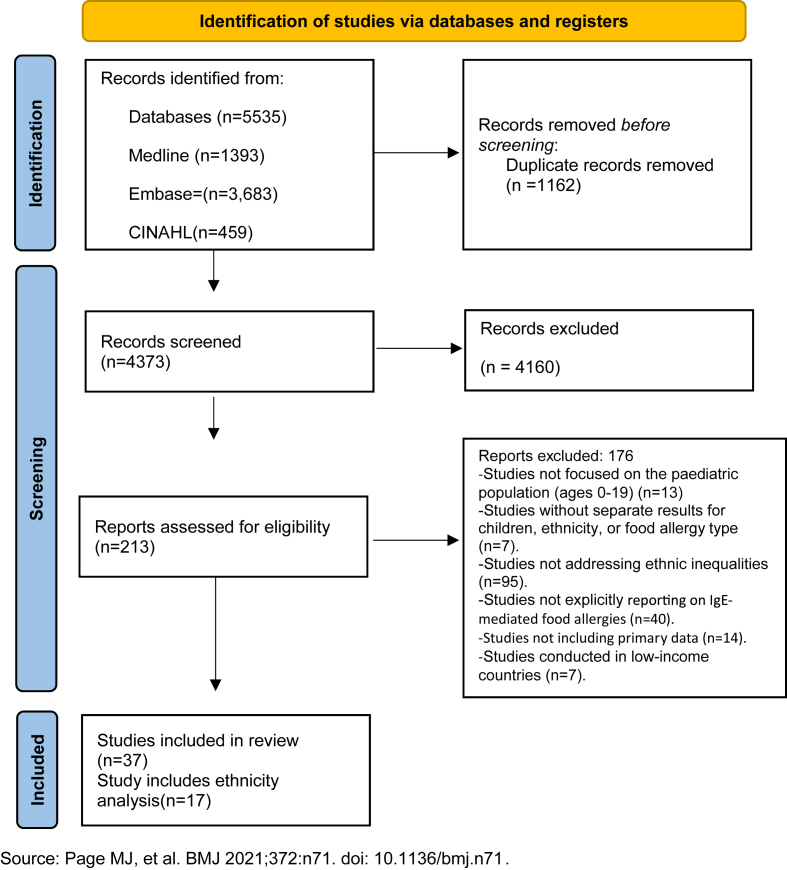
PRISMA flowchart.

After deduplication, 4373 titles and abstracts from the database searches were screened, and 213 were taken forward to full-text screening (Fig. 1). A total of 176 studies were excluded at full-text stage; the remaining 37 included in the final review reported ethnic group data. Twenty of these reported only descriptive ethnic group data in the sample characteristics.^8^^,^^10^^,^^22^, ^23^, ^24^, ^25^, ^26^, ^27^, ^28^, ^29^, ^30^, ^31^, ^32^, ^33^, ^34^, ^35^, ^36^, ^37^, ^38^ whereas 17 studies explicitly reported ethnic group comparisons in their analysis^4^^,^^39^, ^40^, ^41^, ^42^, ^43^, ^44^, ^45^, ^46^, ^47^, ^48^, ^49^, ^50^, ^51^, ^52^, ^53^, ^54^ (Table 2).

### Characteristics of included studies

Detailed characteristics of the included studies are presented in Table 1a, Table 1b. Studies were conducted across different countries, predominantly in the United States (n = 25), the United Kingdom (n = 5), Canada (n = 2), Singapore (n = 2), Australia (n = 1), and New Zealand (n = 1), with 1 study conducted across both the United Kingdom and Japan. Studies utilized diverse observational designs including cross-sectional (n = 11), retrospective (n = 9), prospective cohort (n = 6), longitudinal birth cohort (n = 9), case-control (n = 1), and population-based observational studies (n = 1). Six of the longitudinal cohort papers were derived from 1 study (FA outcomes related to White and African American Racial Differences: FORWARD). No qualitative studies were identified. The publication timeline extended from 2005 to 2024, and the underlying data collection period spanned from 1990 to 2023. There were marked variations in sample size among the studies, with participant numbers ranging from 44 students in a school-based survey^39^ to over 6 million children in a large retrospective cohort study.^25^ The most common allergen investigated in studies, included peanut (n = 24), hen's egg (n = 22), cow's milk (n = 21), tree nuts (n = 18), shellfish (n = 13), wheat, soy and sesame multi-allergen (n = 13), and less common allergens, such as seeds, fruits and vegetables (kiwi, banana, avocado) (n = 2).

**Table 1a:** Overview of included studies reporting ethnicity data without conducting ethnic group analysis

	Dataset source	Country	Data Collection Period	Study aim	Study design	Sample size	Type of food allergy
Amin et al., 2012^22^	Unique	USA	2003 & 2008	To determine the trends in prevalence and clinical characteristics of physician diagnosed pediatric food allergy (FA).	Cross-sectional	2003 = 1852 2008 = 2364	Cow's milk, Eggs, fish, peanuts, sesame, shellfish, soy, tree nuts, wheat
Dyer et al., 2015^23^	Unique	USA	2008–2012	To evaluate trends in ED visits & hospital admissions due to food-induced anaphylaxis among Illinois children and to identify socioeconomic variation in trend distribution.	Retrospective cohort	1893	Peanut, tree nut, fin fish, milk, other food, unknown food
Dyer et al., 2015^24^	Unique	USA	2009–2010	To better characterize peanut allergy prevalence, diagnosis trends, and reaction history among affected children identified from a representative sample of United States households with children.	Cross-sectional	754 peanut allergy 2464 other allergy	Peanut, tree nut, shellfish, fin fish, milk, egg, soy, wheat, and sesame
Feuille et al., 2017^25^	Unique	USA	2007–2013	To assess time trends in food allergy, diagnoses, epinephrine autoinjector (EAI) prescriptions, and EAI administrations in the school setting.	Retrospective cohort	>1 million (6,418,039)	NR
Foong et al., 2024^27^	Unique	UK		To observe changes in PA resolution and persistence over time comparing biomarkers in PA and peanut sensitized but tolerant (PS) children in a population- based cohort.	Longitudinal cohort	947	Peanut
Gupta et al., 2012^26^	Unique	USA	2009–2010	To describe the distribution of childhood allergy in the United States.	Cross-sectional	38,465	Peanut, shellfish, milk, fin fish, Egg, tree nut, wheat, soy
Gupta et al., 2018^10^	Unique	USA	2009–2010	To describe the public health impact of childhood FA by studying a large, nationally representative sample of US households with children.	Cross-sectional	38,408	Peanut, tree nut, Walnut, almond, Hazelnut, Pecan, cashew, pistachio, other tree nut, milk, shellfish, Shrimp, Lobster, Crab, Mollusk, other shellfish, Egg, Fin-fish, wheat, soy, sesame
Jerschow et al., 2014^28^	Unique	USA	1999–2010	To understand the features and time trends in fatal anaphylaxis in the United States from 1999 to 2010.	Retrospective cohort	2458 fatal anaphlaxis cases	NR
Ko et al., 2020^29^	Unique	USA	2016–2018	To describe differences in clinical characteristics in children aged 0–4yrs presenting to the emergency department with food allergy reaction & investigate the association between prehospital epinephrine use & clinical outcomes.	Retrospective cohort	1518	Egg, peanuts, milk product, tree nuts, seed, fish, shellfish, soy, wheat, other, multiple/unclear
Lee et al., 2022^8^	Unique	Singapore	2011–2015	To evaluate the prevalence of cow's milk, egg, and peanut allergies in a general population of Singaporean children below 30 months of age.	Cross-sectional	4115	Cow's milk, Egg, peanut
Nakamura et al., 2025^30^	Unique	Japan and UK	CHIBA: 2010–2011 MAAS: 1995–1997	To evaluate the association between the natural course of egg sensitization and FLG mutations using UK (MAAS) and Japanese (CHIBA) birth cohorts.	Longitudinal cohort	CHIBA n = 285 MAAS n = 1184	Hen's egg white, cow's milk, peanut allergy (MAAS ages 7–8 only)
Samady et al., 2020^31^	Unique	USA	2015–2016	To characterize egg allergy prevalence, severity, baked egg tolerance, and other associated factors in a large US cohort.	Cross-sectional	38,408	Egg
Sasaki et al., 2018^32^	Unique	Australia	2011–2014	To investigate the risk factors for current adolescent food allergy using a population-based sample.	Cross-sectional cluster sample	9816	Peanut, tree nut, egg
Sokol et al., 2020^33^	Unique	USA	NR	To examine the prevalence and manifestations of sesame allergy in a population of US children with IgE-mediated food allergy and to determine the utility of sIgE testing in predicting sesame allergy in this cohort	Prospective cohort	119	Sesame, peanut, tree nut, almond, Brazil nut, cashew, pistachio, Hazelnut, Pecan, Walnut
Speakman et al., 2018^38^	Unique	New Zealand	2006–2015	Investigated the incidence of pediatric (0–14 years) FIA hospital presentations in NZ over a 10-yearperiod	Prospective cohort	1441	Nuts, Cow's milk, Eggs, Seafood, Fruit/Vegetables, wheat/gluten,
Tackett et al., 2019^34^	Unique	USA	2017	To provide a preliminary investigation of food insecurity, perceived risk of food allergen exposure, and the primary food purchase location in a diverse sample of children with food allergy.	Observational	922	NR
Tham et al., 2018^35^	Unique	Singapore	2009–2010	To explore the associations between the timing of allergenic food introduction and the development of food allergy in the GUSTO^72^ birth cohort.	Longitudinal birth cohort study	38,408	Cow's milk, Egg yolk, egg white, peanut, shellfish
Warren et al., 2022^36^	Unique	USA	2015–2016	To leverage data from a large, nationally representative survey sample of US households to characterize the current population-level burden of CMA across the lifespan, while also characterizing clinical and phenotypic differences between individuals with CMA who can and cannot tolerate baked forms of milk.	Cross-sectional	3096	Cow's milk allergy
Ducharme et al., 2022^37^	Unique	Canada	2011–2020	Evaluate the rate, clinical characteristics, and management (prehospital and in the EDs) of TNA in children across Canada	Prospective cohort	540	Tree nuts - almond, brazil, hazel, cashew, macadamia, pecan, pine, pistachio, walnut
Kaman et al., 2023^73^	Unique	USA	2021	To investigate the relationship between OIT and anxiety in children with peanut allergy to help providers and families have an improved shared medical decision discussion around the benefits of initiating OIT.	Prospective, cross-sectional cohort	Control: n = 91 Prescribed OIT: n = 114	Tree nut, egg, milk, wheat, soy, fish, shellfish, sesame

**Table 1b:** Overview of included studies that conducted ethnic group analysis

	Dataset source	Country	Data Collection Period	Study aim	Study design	Sample size	Type of food allergy
Ahmed et al., 2018^39^	Unique	Canada	2015–2016	To investigate the prevalence of asthma, allergic rhinitis, eczema, & allergies among Canadian Inuit children, especially those living in arctic & subarctic areas.	Cross-sectional	44	Peanut, tree nut, egg white
Luyt et al., 2016^40^	Unique	UK	2012–2014	To study the ethnic differences in prevalence of cashew, pistachio & almond allergy	Prospective cohort	2638	Almond, Brazil, cashew, Hazel, peanut, pistachio, Pecan, Walnut, Egg white, milk
Buka et al., 2015^41^	Unique	UK	2012	To determine the incidence & severity of anaphylaxis in the South Asian population & determine the differences between South Asian & white populations	Retrospective cohort	105	All food-induced anaphylaxis, mainly nuts
Dias et al., 2008^42^	Unique	UK	2003–2004	To look at the frequency of different food allergies within a geographically defined UK population, looking specifically at any ethnic variations	Retrospective cohort	76	Egg, peanut, tree nut, Cow's milk, cod, sesame, Kiwi, Pulses (bean, peas and lentils), Banana, Shrimp, avocado, Pumpkin, wheat, Coconut
Fox et al., 2015^43^	Unique	UK	1990–2004	To establish the ethnicity of children with peanut allergy born over a 14-yr period and compare any changes with those among relevant control subjects.	Case control study	420	Peanut, Egg allergy (used as a control)
Gallagher et al., 2024^44^	Unique	USA	2021–2023	To assess disparities in the use of oral immunotherapy in patients with peanut allergy based on race, ethnicity, and socioeconomic status at a single academic medical center.	Retrospective cohort	1028	Peanut
Hannaway et al., 2005^45^	Unique	USA	2003–2004	To analyze the demographics of school children with diverse racial, ethnic & socioeconomic characteristics dispensed injectable epinephrine.	Prospective cohort	181	Nut allergy
Jiang et al., 2023^4^	Unique	USA	2015–2016	To estimate the national distribution of food allergies across racial, ethnic, & socioeconomic groups in the US.	Cross-sectional	38,408	Peanut, milk, shellfish, tree nut, Egg, fin fish, wheat, soy, sesame
Joseph et al., 2021^46^	Unique	USA	2015–2019	To determine if sesame allergy is more prevalent among middle eastern/North African patients in an urban healthcare setting.	Retrospective cohort	14,916 food allergy (140 sesame allergy)	Sesame, other food allergy
Joseph et al., 2016^47^	WHEALS birth cohort	USA	2001–2007	To explore racial differences in IgE-mediated food allergy (IgE-FA) in a birth cohort.	Retrospective cohort	590	Milk, Egg, peanut
Sakai-Bizmark et al., 2019^48^	Unique	USA	2009–2014	To assess urban/rural differences by age and race/ethnicity in emergency department pediatric visit rates for food-induced anaphylaxis, testing the hypothesis that urban residence predicts higher ED visit rates due to food-induced anaphylaxis than rural residence.	Population-based observational study	5850	NR
Brewer et al., 2022^49^	FORWARD cohort	USA	2018–2023	To investigate potential racial differences in parent/caregiver reported timing of infant introduction to peanut, milk, and egg-products among children with allergies to these foods.	longitudinal cohort	632	Peanut, milk, egg
Brown et al., 2021^50^	FORWARD cohort	USA	2018–2023	To evaluate bullying experiences of Black & white children with FA, including associations with peer relationships, anxiety & school policies		252	Peanut, milk, egg, fin fish, shell fish, wheat, tree nuts, sesame, soy, other
Coleman et al., 2022^51^	FORWARD cohort	USA	2018–2023	To examine access to allergen-free foods among Black and white children with FA.		336	Peanut, tree nut, finfish, shellfish, milk, Egg, wheat, soy, sesame
Dileep et al., 2023^52^	FORWARD cohort	USA	2018–2023	To understand if disparities in the prevalence of atopic comorbidities among children with FA are driven by individual and community-level socioeconomic status.^12^		700	NR
Mahdavinia et al., 2022^53^	FORWARD cohort	USA	2018–2023	To investigate the prevalence & associated risk factors of reported gastroesophageal refulx (GER) in children with food allergy.		714	Peanut, tree nut, finfish, shellfish, milk, Egg, wheat, soy, sesame
Mahdavinia et al., 2021^56^	FORWARD cohort	USA	2018–2023	To phenotypic differences such as allergies to different foods and allergic comorbidities between African American & white children with FA enrolled in the FORWARD study.		664	Peanut, milk, Egg, wheat, soy, sesame, tree nuts (>1), fin fish (>1), shellfish (>1)

**Table 2:** Detailed characteristics of studies reporting ethnic group analysis

Author/Year	Ethnic group / Race	Age range	Sex	IgE mediated food allergy diagnosis method	Outcome
Ahmed et al., 2018^39^	Inuit & non-Inuit	6–7 yrs	26 Male 18 Female	Combination of Hx of self-reported & skin prick	Identify the prevalence of asthma, allergic rhinitis, eczema, & allergies among Canadian Inuit children.
Luyt et al., 2016^40^	South Asian & white	<18yrs	N/A	Physician diagnosed SPT	Identify the prevalence of an allergy to nuts, cow's milk & eggs in south Asian compared to white children.
Buka et al., 2015^41^	South Asian, another ethnicity vs white	<16	59 Male 46 Female	Physician diagnosed anaphylaxis	Determine the incidence and severity of anaphylaxis in this population and to investigate the differences between the South Asian and white populations.
Dias et al., 2008^42^	Caucasian, Non-Caucasian) Asian/Asian British, Black/Black British & other)	6mo - 13.5yrs	40 Males 36 Females	Physician diagnosed SPT with history	Identify any ethnic deference's between the frequency of different food allergies within a geographically defined UK population.
Fox et al., 2015^43^	Non white (Asian Indian/Pakistani, Black-African/Arab, Asian Chinese, other, mixed) vs white	<18yrs	N/A	1.Convincing history of peanut allergy together with a positive skin prick or specific IgE test 2.+ve oral food challenge 3.a specific IgE level of greater than 15 kU/L or an SPT response of greater than 7 mm in the absence of any history of peanut exposure.	Examine changes in the proportion of children from nonwhite ethnic backgrounds diagnosed with peanut allergy over a 14-year period.
Gallagher et al., 2024^44^	American Indian, Asian, Black, Hawaiian, other, patient refuse, unknown vs white	<18yrs	N/A	International statistical classification of diseases and Related Health ICD codes: allergy to peanuts and anaphylactic reaction due to peanuts	Explore potential disparities in race, ethnicity, and socioeconomic status among the patient group undergoing OIT in a single center that cares for a broad range of individuals with peanut allergies
Hannaway et al., 2005^45^	Hispanic, Black, Asian, other vs white	4yrs–18yrs	69 Males 49 Females	Self-reported allergy and epinephrine prescription	Identify demogrpahics of schoolchildren with dispensed injectable epinephrine.
Jiang et al., 2023^4^	Multible Asian, Black, Hispanic, white & multiple& other	All ages	Male 5439 Females 4287	1.Parent-reported convincing FAs 2.Physician diagnosed	The prevalence of food allergies and their clinical outcomes, such as emergency department visits, epinephrine autoinjector use and severe reactions.
Joseph et al., 2021^46^	White, Black, Hispanic, other & unknown	0yrs–18yrs	7603 Male, 7453 Female	1.Physician diagnosed IgE by specifc IgE >0.35 IU/mL 2.Epinephrine prescription	Is sesame allergy more prevalent among middle eastern/North African patients in an urban healthcare setting.
Joseph et al., 2016^47^	Black & all other	0–36 months	312 Male 278 Female	Physician diagnosed by skin prick tests	Determine racial differences in IgE-mediated food allergy in a birth corhort.
Sakai-Bizmark et al., 2019^48^	White, Black & other	<18 yrs	3462 Male 2388 Female	Food-induced anaphylactic reaction based on (ICD-9-CM)	Incidence of food induced anaphylaxis in rural and urban areas by age group (0–4yrs, 5–9yrs, 10–14yrs, 15–17yrs), race groups, sex and insurance
Brewer et al., 2022^49^	Black & White	<6mo, 7-11mo & >11mo	242 Male 394 Female	Allergist-diagnosed positive food-specific IgE or skin prick test	Identify potential racial differences in parent/caregiver reported timing of infant introduction to peanut, milk, and egg-products amoung children with allergies to these foods.
Brown et al., 2021^50^	Black & White	<12yrs	157 Male 59 Female	Allergist-diagnosed positive food-specific IgE or skin prick test	Identify bullying experiences of Black & White children with FA, including associations with peer relationships, anxiety & school policies
Coleman et al., 2022^51^	Black & White	<12yrs	222 Males 114 Females	Allergist-diagnosed positive food-specific IgE or skin prick test	Identify if Black children have less access to allergen-free foods than White children
Dileep et al., 2023^52^	Non-Hispanic White Non-Hispanic Black Hispanic/Latinx	4 and 12 years	388 Males 236 Females	Allergist-diagnosed positive food-specific IgE or skin prick test	To identify any disparities in the prevalence of atopic comorbidities among food-allergic children.
Mahdavinia et al., 2022^53^	Black & White	<12yrs	N/A	Allergist-diagnosed positive food-specific IgE or skin prick test	Identify the prevalence & associated risk factors of reported gastroesophageal refulx (GER) in children with food allergy.
Mahdavinia et al., 2021^56^	Black & White	<12yrs	411 Male, 245 Female	Allergist-diagnosed positive food-specific IgE or skin prick test	Characterise food allergen profiles & associated comorbidities of African American & White children.

### Results of studies reporting ethnic group analysis

Detailed characteristics of studies with ethnic group analysis are presented in Table 2. These studies used a range of approaches to categorize ethnicity. Some studies relied on binary comparisons such as Black versus White,^49^, ^50^, ^51^^,^^53^^,^^55^^,^^56^ which may reduce analytical precision by aggregating multiple heterogeneous ethnicities into a single category, obscuring meaningful differences between populations. Other studies included broader categories, such as White, Black, Asian, and Hispanic, offering clearer comparisons but still assuming internal homogeneity within each group.^4^^,^^32^^,^^39^, ^40^, ^41^, ^42^, ^43^, ^44^, ^45^, ^46^, ^47^

White populations were most frequently reported as the comparison group, followed by the Black populations, and Asian populations. Several studies reported Asian speciifc subgroups such as South Asian, British Asian, and Indian, Pakistani, and Chinese. Additional ethnic groups included Inuit and non-Inuit, American Indian, Arab, and Hawaiian. Some studies classified participants into multiple/mixed and other/unknown ethnic groups. Ethnic group analysis focused on prevalence of IgE-mediated FA and atopic comorbidities (n = 9), access to care and management (n = 3), and health outcome severity (n = 5).

Diagnostic criteria used in studies for IgE-mediated FA diagnosis, included skin prick test or a specific IgE blood test (n = 9), parent-reported clinical history using strict diagnostic criteria (n = 4), the gold-standard graded oral food challenge (n = 1), a combination of diagnostic criteria (n = 1), and International Classification of Diseases (ICD) codes for food-induced anaphylaxis or IgE-mediated peanut allergy (n = 2).

### Prevalence differences by ethnicity

Nine studies reported prevalence differences by ethnicity (Appendix 5a).

### Black participants

Six studies, conducted predominantly in the United StatesA with 1 study from the United Kingdom, included analyses relating to Black children. Brewer et al^49^ found that the most prevalent allergy was peanut allergy, affecting 413/632 (63%) of cohort with similar prevalence between Black children 153/234 (65.4%) and White children 260/398 (65.3%). Black children had delayed introduction of peanut and milk compared with White children. White children were significantly more likely to have early introduction of peanut (OR 2.6; 95% CI: 1.1–7.2) and cow's milk (OR 2.7; 95% CI: 1.1–6.7) at <6 months, and less likely to have delayed introduction of peanut (OR 0.13; 95% CI: 0.1–0.5) and cow's milk (OR 0.2; 95% CI: 0.1–0.6) >11 months. Moreover, nearly 89% of Black children were not introduced to peanut before 1 year of age, compared with 67% of White children. Jiang et al^4^ reported that American Black children had the highest overall prevalence rates of convincing IgE-mediated FA (8.9%; 95% CI: 7.6%–10.3%) and convincing peanut allergy (3.0%; 95% CI: 2.4%–3.8%), compared to children with other race/ethnicities. They also reported the highest rates for hen's egg allergy (1.6%; 95% CI: 1.0%–2.7%) and finfish allergy (0.9%; 95%CI:, 0.6%–1.5%) among Black children. Similarly, Joseph et al^47^ found a high proportion of African American children with peanut allergy, although this difference was not statistically significant (p = 0.75). However, when examining sensitization where serum specific IgE was >0.35 IU/mL, prevalence was significantly higher among African American compared with non-African American children (43.2% vs 32.3%, p = 0.01). In terms of peanut allergy, African American children had a 1.7% prevalence of IgE levels greater than 95% predictive decision points, compared to 0.5% for non-African American children; however, this difference was not statistically significant (p = 0.43). African American children were significantly more likely to be sensitized to multiple allergens including (milk, egg, and peanut) (AOR 1.80; 95% CI 1.22–2.65). Likewise, Mahdavinia et al^54^ reported that African American children had significantly higher adjusted odds of finfish (AOR 2.54; p < 0.01) and shellfish (AOR 3.10; p < 0.001) allergies compared with White children. In contrast, both Joseph et al^46^ and Mahdavinia et al^54^ found that Black children were less likely to have sesame allergy. Fox et al^43^ found that the proportion of Black African/Arab children diagnosed with peanut allergy decreased slightly from 15.7% in 1990–1998 to 14.5% in 1999–2005 periods. For hen's egg allergy within the same group, the proportion was 32/64 (22.2%) in 1990–1998 and 45/105 (19.7%) in 1999–2005 periods, both showing no statistically significant change.

### Asian populations

Three studies, including 2 conducted in the United Kingdom and 1 in the United States, included analyses relating to Asian children. Luyt et al^40^ reported that in the United Kingdom, South Asian children had a significantly elevated risk of allergy to specific type of tree nuts, with a relative risk of almond allergy of 3.95 (95% CI: 1.43–10.89) compared to White children. Similarly, cashew nut relative risk for allergy was significantly increased for South Asian children compared with White children (RR 2.59; 95% CI: 1.68–4.00) and for pistachio nut allergy (RR 3.71; 95% CI: 2.23–6.16). However, increased risk for peanut or other food allergies was not found. Conversely, Fox et al^43^ in the United Kingdom reported a statistically significant increase in the prevalence of peanut allergy among South Asian (5.99%; p = 0.016) and Chinese children (3.39%; p = 0.013) alongside a decrease among White children (−23.49%) over the same period. Recently, a US study (Jiang et al^4^) reported that Asian children had the lowest prevalence of convincing IgE-mediated FA (6.5%; 95% CI: 5.1–8.2%), compared with children from all other ethnic groups. However, they found that Asian children had a higher prevalence of developing tree nut allergy (2.0%; 95% CI: 1.2%–3.2%) compared with other ethnic groups.

### Other ethnic or cultural classifications

Three studies, conducted in the United States, Canada, and the United Kingdom included analyses relating to other ethnic or cultural classifications, including Middle Eastern/North African (MENA), Inuit and non-Inuit populations, non-White groups, and children of unknown ethnicity. Joseph et al^46^ in the United States found that children from MENA backgrounds had higher odds of sesame allergy compared with children from all other ethnic groups combined (OR 3.15; 95% CI: 1.85–5.08). Children with unknown ethnicity also had higher odds of sesame allergy when compared with non-Hispanic White children, although this association was not statistically significant (OR 1.26; 95% CI: 0.80–1.94). In Canada, Ahmed et al^39^ reported that non-Inuit children had a higher prevalence of FA compared with Inuit children. However, the study sample was small (n = 44). Hen's egg white allergy was reported among 3.3% of participants, with 1 case among non-Inuit group, while peanut allergy prevalence was 6.7%, with 1 case in the Inuit group. In the United Kingdom, Dias et al^42^ found that non-White children had significantly higher average number of FAs per child (2.05), compared with White children (1.22), with a mean difference of 0.83 (p < 0.01). Furthermore, non-White children were over-represented in the allergy clinics (52.6% compared to 35.9% of the general pediatric population and 21% of the local population), indicating a mean disparity of 16.7% (p < 0.01). Also, FA reaction was earlier among non-White children (1.7 years, range 0.3–8 years) compared to White children (2.6 years, range 0.3–12 years: p < 0.05).

### Access to care and management

Access to care and management was reported by 3 studies relating to oral immunotherapy uptake,^44^ access to epinephrine autoinjectors,^45^ and access to allergen-free foods^51^ (Appendix 5b). In a US retrospective cohort study, Gallagher et al^44^ reported that Black children were underrepresented in oral immunotherapy (OIT), accounting for 4.1% of those receiving OIT, compared with 18% of peanut-allergic control group (p < 0.0001). White children were overrepresented, comprising 75% of the OIT group compared with 63.6% of controls (p = 0.0068), while Asian children had similar representation across groups. Similarly, in the United States Hannaway et al^45^ examined 3 Massachusetts school districts and found clear differences in dispensing of injectable epinephrine. White students were significantly more likely to receive an epinephrine autoinjector than non-White students (OR 4.5; 95% CI: 2.75–7.36). The highest rate was seen among White students in an affluent suburban district (1.23%), whereas there was a much lower rate among minority students in the urban district (0.17%). Asian and Hispanic students in Lynn (urban district) had the lowest dispensing rates (0.14% and 0.10% respectively). Coleman et al^51^ highlighted inequalities in access to allergen-free foods. White caregivers were significantly more likely than Black caregivers to report availability of these products (88.1% vs 59%, p < 0.001) and to purchase allergen-free foods online (35.2% vs 12%, p < 0.001).

### Health outcome severity

The health outcomes reported by 5 studies related to anaphylaxis,^32^^,^^41^ psychosocial outcomes,^50^ and atopic and clinical comorbidities^53^^,^^55^ (Appendix 5c). Sakai-Bizmark et al^48^ in the United States examined the incidence of food-induced anaphylaxis in different populations. They observed that Black children had the highest emergency department visits at 15.26 per 100,000, compared with 10.00 per 100,000 among non-Hispanic White children and 9.21 per 100,000 among other races. In rural areas, the incidence of anaphylaxis increased in older ages for all racial groups, whereas the highest incidence rates were observed among younger children in urban areas.

Brown et al^50^ examined psychosocial outcomes among Black and White school-aged children with FA in a US cohort. Around 20% of children experienced FA-related bullying, and there were no significant racial differences in the overall prevalence. For children aged 11 years and older, bullying was reported more often by White parents than Black parents (18.2% vs. 0%; p = 0.046). Most measures of peer interaction and anxiety showed no significant racial differences. However, Black parents reported higher scores for anxiety-related items, including "my child felt nervous" and "my child felt worried" (p = 0.02). White parents more frequently reported access to allergen-free lunch areas at school compared with Black parents (48.1% vs. 19.1%; p = 0.006).

In relation to atopic comorbidities, Dileep et al^55^ found that Black children had significantly higher likelihood of asthma (OR 2.76; 95% CI: 1.77–4.29) and allergic rhinitis (OR 2.50; 95% CI: 1.63–3.85) than White children. Mahdavinia et al^53^ in the United States examined gastroesophageal reflux (GER) and found similar rates between Black and White children. However, Black children were less likely to receive treatment for GER with 43.6% untreated compared with 18.1% of White children (p = 0.002). Among White children, GER was associated with a higher prevalence of milk allergy (41.5% among those with GER vs 20.6% among those without GER), soy allergy (9.6% vs 4.1% respectively), and multiple FAs (74.5% vs. 60.4% respectively) (p < 0.05), and a lower prevalence of tree nut allergy (41.5% vs. 53.3% respectively) (p < 0.05). Black children with GER were also more likely to have eczema (p < 0.05).^53^ Buka et al^41^ in the United Kingdom showed that 87.5% of South Asian children experienced food-induced anaphylaxis, a proportion almost identical to that observed among White children (87%). Among South Asian children, 37.5% of food-related reactions were triggered by nuts, compared with 56.5% in White children. The study reported no statistically significant ethnic differences in the distribution of food triggers; however, the sample size was limited (n = 38) therefore results need to be interpreted with caution.

## Discussion

This scoping review aimed to explore the existing evidence on ethnic group health inequalities in paediatric IgE-mediated FA conducted in HICs. Most studies identified focused on prevalence differences between ethnic groups, and were conducted in the United States and United Kingdom, with fewer studies originating from Canada, Singapore, Japan, Australia, and New Zealand, and no studies were identified from other HIC contexts. Among the articles that reported evidence from the United States, many were from the FORWARD study which reduces the context variability of the populations from which observations were available. It also reflects an imbalance in FA and ethnicity research, a limitation noted by Sicherer and Sampson,^57^ who reported that racial and ethnic disparities remain poorly characterized and inconsistently measured in FA research.^57^

There was substantial heterogeneity between studies included in this review, particularly related to categorization of ethnic groups and limited focus on exploring ethnic group differences in access to care and health outcomes. Further research is a need on ethnic differences in IgE mediated allergy in a wider range of contexts to understand where inequalities are and how services can address them. This review has also highlighted a dearth of qualitative research. Qualitative research is particularly important, especially among different ethnic groups, as it enables the exploration of patients lived experiences and distinct needs. The absence of, and pressing need for, qualitative studies has also been emphasized in recent work focusing on British South Asian adult populations.^58^

The most frequent diagnostic approaches used in studies were skin prick tests and specific IgE serum measurements, which are widely used as the first-line diagnostic tools in clinical settings.^2^ These methods are readily available and inexpensive; however, they are not a definitive indicator of clinical reactivity. Oral food challenges were used in only a small number of studies, despite being the gold standard for confirming or excluding FA, likely due to their time-consuming, resource-intensive, and complex nature.^2^

The terminology used to define and record ethnic groups varied across the studies, reflecting an issue that is consistently highlighted in healthcare research.^59^ This variability illustrates the socially constructed nature of ethnicity, and the differing census and institutional classifications adopted in each country. Across the included studies, ethnicity was self-reported, which is standard practice in health research. People vary in describing their ethnicity, as ethnicity reflects social identity and lived experience. For some, it is personal, and therefore, they do not wish to share this information. The categories such as Other, Unknown, Multiple/Mixed, or Patient Refused were noted in studies; these broad and overlapping groupings may hide important differences within groups and reduce the quality and interpretability of ethnicity data. JAMA recommendations also advise authors to be specific when reporting on racial and ethnic groups.^59^ These recommendations highlight that, although race, ethnicity, and ancestry are often used synonymously, they represent distinct concepts within health research.^59^ Researchers should consider which term is appropriate for their study context, provide explicit definitions, and apply them consistently throughout their work, and the classification should be self-reported rather than assigned by an observer.^60^ Some studies also fail to distinguish clearly between race and ethnicity or collapse them into a single construct, which can obscure important health-related differences. The lack of definitions or justifications for terms like “Caucasian,” “European American,” or “Hispanic” further complicates the interpretation of findings. This ambiguities may mask within-group diversity and intersecting identities, potentially misrepresenting health inequalities, particularly in racially diverse populations. Similar concerns have been raised about the imprecise use of racial and ethnic categories, which can have significant implications for the accuracy and relevance of research outcomes.^61^

Most included studies were focused on prevalence of FA by ethnicity, and overall, the findings suggest increasing trends affecting different minoritized ethnic groups. Our review supported the conclusion of another similar review in relation to observation of a greater occurrence of FAs among Black children in the United States.^36^^,^^62^ Five studies specifically analyzed data for Black or African American populations, which indicated that these groups experienced a greater burden of FAs. They had a higher rate of peanut allergy, more sensitized to multiple FAs, and had higher odds of fish and finfish allergies compared with other groups. Researchers attributed differences to factors such as socioeconomic status, health care accessibility, and historical circumstances faced by specific population groups.^36^

We noted that peanut allergy is not consistently elevated in Asian populations; however, tree nut allergy was a more consistent risk factor across studies. Interestingly, some studies indicate a rise in peanut allergy in Asian minorities living in Western countries, although the overall prevalence remains lower than White populations. Conversely, studies conducted within Asian regions indicates that shellfish allergy is highly prevalent, whereas peanut and tree-nut allergies predominated in Western countries.^63^^,^^64^ The reason for these differences in peanut allergy remains uncertain; however, dietary and cultural practices may play a role.^65^ In many Asian countries, peanuts are commonly consumed in boiled, braised, or cooked forms, which reduces allergenicity and may promote the development of immune tolerance.^65^ In contrast, Western diets often favor roasted peanuts, a process known to increase allergenic potency. These cultural patterns of preparation and introduction may contribute to the observed regional differences in peanut allergy prevalence. Supporting this, a survey conducted in Singapore with participants age ranging from 1 to 58 years found that individuals born in Asia had a lower risk of peanut and tree nut allergies compared to those born in Western countries, irrespective of their ethnicity.^66^ Although allergen profiles differ across regions, current allergy guidelines remain heavenly focused on peanut allergy, and do not adequately address different allergens variations. These gaps highlight the need for culturally tailored prevention and management strategies to ensure that they are applicable across different populations.

In our review, evidence on health outcome severity highlights consistent inequalities with higher burden of FAs borne by Black children. Black children had the highest rate of emergency department visits for food-induced anaphylaxis in urban areas compared with White children and other racial groups. Current British Society for Allergy and Clinical Immunology (BSACI) guidelines emphasized the importance of prescribing adrenaline autoinjector for those at risk of anaphylaxis to improve outcomes.^67^ Yet, studies show that children from ethnic minority groups are less likely to be prescribed epinephrine,^45^ despite increases in the overall rates of anaphylaxis some studies reported no significant differences in the rates of food-induced anaphylaxis between White and non-White children.^23^^,^^68^

The position statement by American Academy of Allergy, Asthma and Immunology (AAAAI) also reported that ethnic and racial minorities bear a disproportionately higher burden and more severe outcomes of FAs in the United States.^69^ Structural inequities including limited access to allergen-free foods, higher emergency care costs, poor housing conditions, and lower prescription rates exacerbate these inequalities.^69^ Moreover, underrepresentation of minoritized groups in clinical research reduces the generalizability of findings and restricts access to medical advances. According to Anagnostou et al^69^ addressing these disparities demands a focus on social determinants of health, diversity in research participation, and equitable access to allergy care to reduce the burden faced by minorities.

Beyond anaphylaxis, similar inequalities were observed in other comorbidities. For example, 1 study reported that, despite similar rates of GER between Black and White children, Black children were less likely to receive treatment and had higher prevalence of eczema.^53^ Furthermore, prevalence of FA comorbidities, such as asthma and allergic rhinitis, was significantly higher amongst Black children. This aligns with wider literature showing that Black children experience higher prevalence of asthma hospitalizations and mortality.^70^

### Strengths and limitations

A systematic, rigorous, and highly sensitive process in developing and refining the search strategy was used to ensure the application of robust and comprehensive searches to identify relevant data. The use of both controlled vocabulary and free-text keywords ensured that the search was comprehensive across multiple databases. All screening and data extraction were carried out in duplicate ensuring rigor and reliability in the review process. Our findings are novel as there are no comparable published HIC-based reviews that explore evidence for ethnic group inequalities in pediatric IgE mediated FAs. Restricting the review to HICs allowed exploration of patterns of ethnic health inequalities within broadly comparable healthcare systems. However, it remains important to explore whether similar patterns are observed in low- and middle-income settings, particularly in locations with different ethnic group population size and distribution.

A scoping review approach was selected as the most appropriate design to address the broad aim of this research, which sought to map and synthesize the extent and nature of existing evidence on ethnic group inequalities in pediatric IgE-mediated FAs. This approach is particularly suitable for areas where evidence is heterogeneous and emerging, allowing for a comprehensive overview of study characteristics, concepts, and gaps rather than an assessment of intervention effectiveness. As recommended by the JBI and PRISMA-ScR guidance, scoping reviews do not include a formal quality appraisal or risk-of-bias assessment, as their purpose is to provide an overview of the available evidence rather than to evaluate the strength or direction of effects.^16^^,^^71^ This review identified important evidence gaps and methodological limitations within the current literature. Although ethnic groups were commonly reported, relatively few studies examined this analytically, suggesting that available datasets were not fully utilized to explore ethnic differences in pediatric IgE-mediated FA. Much of the existing evidence also derives from a limited number of cohort studies, which constrains the diversity of populations and healthcare settings represented. The lack of qualitative research represents a notable gap, particularly regarding family experiences, cultural influences, and access to allergy care among different ethnic groups. Incorporating qualitative methods alongside quantitative approaches would enhance understanding of the social and contextual factors underlying observed inequalities. Race and ethnicity were also defined and categorized inconsistently across studies. Broader classifications such as “non-White” or “Asian” combine heterogeneous populations and limit comparability. Future research should adopt standardized definitions of ethnic groups to improve data quality and enable meaningful comparisons.

Addressing these gaps through inclusive, mixed-methods, and clearly defined research designs would strengthen the evidence base and contribute to a more comprehensive understanding of ethnic group inequalities in pediatric Ige mediated FA.

## Conclusion

Current evidence suggests that children across different ethnic groups with IgE-mediated FA experience differences in prevalence, access to care and management, and health outcomes. Large-scale research is needed to identify the underlying factors that contribute to inequalities in access to FA diagnosis and treatments. Further research is needed to inform the development of allergy services to ensure that all children have equitable access to care and health outcomes, regardless of ethnic group or race.

## Ethics statement

The study was considered exempt from ethics because it was a review of the literature and did not involve human subjects.

## Author contributions

BA, NH, AF and LM were involved in the conception and design of the review including methodological framework. BA conducted literature searches and BH, NH, AF, EP, LM and MP LM involved in screening. BH, EP and SH involved in data extraction. BA led the analysis, with support from NH and MP. BA prepared the manuscript. All authors contributed to reviewing and editing the manuscript.

## Submission declaration

This manuscript has not been published previously and is not under consideration elsewhere.

## Disclosure of generative AI and AI-assisted technologies

During the preparation of this manuscript, the authors used (ChatGPT, OpenAI) to assist with English grammar checking and language refinement. The authors reviewed and edited the content and take full responsibility for the final manuscript.

## Financial disclosure

This research was supported by a PhD studentship funded by the Saudi Cultural Bureau (1079049472).

## Competing interest

The authors declare no competing interests. LJM has previous national commercial clinical trials with associated Advisory Boards/Lectures with Danone, ALK and Regeneron. LJM is Secretary (Immunotherapies Registry) and Vice President (Services) for the British Society of Allergy and Immunology, United Kingdom. LJM has published with the National Health Service Business Services Authority.
