# Supplementary material for: Pediatric IgE mediated food allergies and ethnic group inequalities: A scoping review
Source: World Allergy Organ J. 2026 May 12;19(6):101395. doi: 10.1016/j.waojou.2026.101395 (PMC13195539; doi:10.1016/j.waojou.2026.101395)
Supplement: Multimedia component 1 [file mmc1.docx]

**Appendix 1.** Key words derived from PCC elements that were used in the search strategy

| OR | AND | | | | |
| --- | --- | --- | --- | --- | --- |
|  | **Population** | **Concept** | **Concept 2** | **Concept 3** | **Context** |
|  | Paediatric  Children  Infant  Babies  Toddler  School children  Adolescent  Teen  Youth  Juvenile | Food allergy  Food Hypersensitivity  IgE allergy  Anaphylaxis | Health inequality  Health equity  Demography  Differences | Ethnic group  Ethnicity  Racial  Culture  Minority  African  Black  Asian  Hispanic  Middle easterner | High income country |

**Appendix 2.** High-income countries list as classified by the World Bank

| Country / territory | Country / territory | Country / territory |
| --- | --- | --- |
| American Samoa | Iceland | Portugal |
| Andorra | Ireland | Puerto Rico |
| Antigua and Barbuda | Isle of Man | Qatar |
| Aruba | Israel | Romania |
| Australia | Italy | Russian Federation |
| Austria | Japan | San Marino |
| Bahamas, The | Korea, Rep. | Saudi Arabia |
| Bahrain | Kuwait | Seychelles |
| Barbados | Latvia | Singapore |
| Belgium | Liechtenstein | Sint Maarten (Dutch part) |
| Bermuda | Lithuania | Slovak Republic |
| British Virgin Islands | Luxembourg | Slovenia |
| Brunei Darussalam | Macao SAR, China | Spain |
| Bulgaria | Malta | St. Kitts and Nevis |
| Canada | Monaco | St. Martin (French part) |
| Cayman Islands | Nauru | Sweden |
| Channel Islands | Netherlands | Switzerland |
| Chile | New Caledonia | Taiwan, China |
| Croatia | New Zealand | Trinidad and Tobago |
| Curaçao | Northern Mariana Islands | Turks and Caicos Islands |
| Cyprus | Norway | United Arab Emirates |
| Czechia | Oman | United Kingdom |
| Denmark | Palau | United States |
| Estonia | Panama | Uruguay |
| Faroe Islands | Poland | Virgin Islands |
| Finland |  |  |
| France |  |  |
| French Polynesia |  |  |
| Germany |  |  |
| Gibraltar |  |  |
| Greece |  |  |
| Greenland |  |  |
| Guam |  |  |
| Guyana |  |  |
| Hong Kong SAR, China |  |  |
| Hungary |  |  |

**Appendix 3.** Non IgE-mediated and mixed IgE mediated food allergies

| **Non-IgE mediated food allergy**   - Contact dermatitis - Food protein-induced enterocolitis syndrome (FPIES) - Food protein-induced allergic proctitis and proctocolitis - Food protein-induced enteropathy - Dermatitis herpetiformis - Coeliac disease | **Mixed IgE and non-IgE mediated food allergy**   - Exacerbation of atopic eczema without a clinical history of IgE mediated food allergy - Eosinophilic oesophagitis - Eosinophilic gastritis/enteritis - Exacerbation of asthma due to the environment aeroallergens and NOT associated to foods |
| --- | --- |

**Appendix 4.** Search strategy

Medline

| 1. | pediatrics/ or pediatric emergency medicine/ |
| --- | --- |
| 2. | child/ or child, preschool/ or infant/ |
| 3. | P?ediatric*.ti,ab,kf. |
| 4. | child*.ti,ab,kf. |
| 5. | baby.ti,ab,kf. |
| 6. | babies.ti,ab,kf. |
| 7. | toddler*.ti,ab,kf. |
| 8. | "school children".ti,ab,kf. |
| 9. | Adolescent/ |
| 10. | teen*.ti,ab,kf. |
| 11. | adolescen*.ti,ab,kf. |
| 12. | youth.ti,ab,kf. |
| 13. | juvenile*.ti,ab,kf. |
| 14. | pre teen.mp. |
| 15. | preschool.mp. |
| 16. | preteen.mp. |
| 17. | pupil*.mp. |
| 18. | Young Adult/ |
| 19. | Humans/ |
| 20. | or/1-19 |
| 21. | exp Food Hypersensitivity/ |
| 22. | Hypersensitivity/ |
| 23. | Immunoglobulin E/ |
| 24. | IgE mediated food allergy.mp. |
| 25. | food allerg*.ti,ab,kf. |
| 26. | Anaphylaxis/ |
| 27. | Oral allergy syndrome.mp. |
| 28. | food pollen syndrome.mp. [mp=title, book title, abstract, original title, name of substance word, subject heading word, floating sub-heading word, keyword heading word, organism supplementary concept word, protocol supplementary concept word, rare disease supplementary concept word, unique identifier, synonyms, population supplementary concept word, anatomy supplementary concept word] |
| 29. | Food-induced anaphylaxis.mp. |
| 30. | Milk Hypersensitivity/ |
| 31. | Egg Hypersensitivity/ |
| 32. | Nut Hypersensitivity/ |
| 33. | Peanut Hypersensitivity/ |
| 34. | Pea allergy.mp. |
| 35. | Chickpea allergy.mp. |
| 36. | Lentil allergy.mp. |
| 37. | Wheat Hypersensitivity/ |
| 38. | Lupin allergy.mp. |
| 39. | Soya allergy.mp. |
| 40. | Kiwi allergy.mp. |
| 41. | Rice allergy.mp. |
| 42. | Buckwheat allergy.mp. |
| 43. | Mustard allergy.mp. |
| 44. | poppy seed allergy.mp. |
| 45. | sunflower seed allergy.mp. |
| 46. | pumpkin seed allergy.mp. |
| 47. | ("Tree nut allergy" or "Hazelnut allergy" or "Legumes allergy" or "Almond allergy" or "Cashew allergy" or "Chestnut allergy" or "Pistachio allergy" or "Walnut allergy").mp. [mp=title, book title, abstract, original title, name of substance word, subject heading word, floating sub-heading word, keyword heading word, organism supplementary concept word, protocol supplementary concept word, rare disease supplementary concept word, unique identifier, synonyms, population supplementary concept word, anatomy supplementary concept word] |
| 48. | (Sesame Allergy or Pecan allergy or Brazil nut allergy or Soy allergy or Fish allergy).mp. [mp=title, book title, abstract, original title, name of substance word, subject heading word, floating sub-heading word, keyword heading word, organism supplementary concept word, protocol supplementary concept word, rare disease supplementary concept word, unique identifier, synonyms, population supplementary concept word, anatomy supplementary concept word] |
| 49. | (Seafood allergy or Shellfish allergy).mp. [mp=title, book title, abstract, original title, name of substance word, subject heading word, floating sub-heading word, keyword heading word, organism supplementary concept word, protocol supplementary concept word, rare disease supplementary concept word, unique identifier, synonyms, population supplementary concept word, anatomy supplementary concept word] |
| 50. | (Food adj3 anaphylaxis).mp. [mp=title, book title, abstract, original title, name of substance word, subject heading word, floating sub-heading word, keyword heading word, organism supplementary concept word, protocol supplementary concept word, rare disease supplementary concept word, unique identifier, synonyms, population supplementary concept word, anatomy supplementary concept word] |
| 51. | or/21-50 |
| 52. | health inequities/ or healthcare disparities/ or socioeconomic disparities in health/ |
| 53. | health inequalit*.mp. |
| 54. | health equity/ or right to health/ |
| 55. | Demography/ |
| 56. | health equality.mp. |
| 57. | differences.mp. |
| 58. | History, 20th Century/ |
| 59. | Prevalence/ |
| 60. | Public Health/ |
| 61. | or/52-60 |
| 62. | "Ethnic and Racial Minorities"/ |
| 63. | Racial Groups/ |
| 64. | *Ethnicity/ |
| 65. | ethnic*.mp. |
| 66. | population groups/ |
| 67. | exp ethnicity/ or exp racial groups/ |
| 68. | ethnic group*.mp. |
| 69. | racial group*.mp. |
| 70. | race.ti,ab,kf. |
| 71. | sociodemographic factors/ |
| 72. | Culture/ |
| 73. | culture.mp. |
| 74. | Cultural Characteristics/ |
| 75. | characteristic*.mp. |
| 76. | ethnic.mp. |
| 77. | Minority Groups/ |
| 78. | minority group*.mp. |
| 79. | African.mp. or African People/ |
| 80. | black.mp. |
| 81. | Asian.mp. or Asian People/ |
| 82. | Hispanic.mp. or "Hispanic or Latino"/ |
| 83. | Middle Easterner.mp. or Middle Eastern People/ |
| 84. | 62 or 63 or 64 or 65 or 66 or 67 or 68 or 69 or 70 or 71 or 72 or 73 or 74 or 75 or 76 or 77 or 78 or 79 or 81 or 82 or 83 |
| 85. | 20 and 51 and 61 |
| 86. | 84 and 85 |

**Embase**

| 1. | pediatrics/ or pediatric emergency medicine/ |
| --- | --- |
| 2. | child/ or preschool child/ or school child/ or infant/ or child nutrition.mp. or infant nutrition/ [mp=title, book title, abstract, original title, name of substance word, subject heading word, floating sub-heading word, keyword heading word, organism supplementary concept word, protocol supplementary concept word, rare disease supplementary concept word, unique identifier, synonyms, population supplementary concept word, anatomy supplementary concept word] |
| 3. | P?ediatric*.ti,ab,kf. |
| 4. | child*.ti,ab,kf. |
| 5. | baby.ti,ab,kf. |
| 6. | babies.ti,ab,kf. |
| 7. | toddler*.ti,ab,kf. |
| 8. | "school children".ti,ab,kf. |
| 9. | Adolescent/ or adolescent nutrition/ |
| 10. | teen*.ti,ab,kf. |
| 11. | adolescen*.ti,ab,kf. |
| 12. | youth.ti,ab,kf. |
| 13. | juvenile*.ti,ab,kf. |
| 14. | pre teen.mp. |
| 15. | preschool.mp. |
| 16. | preteen.mp. |
| 17. | pupil*.mp. |
| 18. | Young Adult/ |
| 19. | Humans/ |
| 20. | or/1-19 |
| 21. | exp food allergy/ |
| 22. | immediate type hypersensitivity/ |
| 23. | immunoglobulin E/ or immunoglobulin E.mp. |
| 24. | IgE mediated food allergy.mp. |
| 25. | food allerg*.ti,ab,kf. |
| 26. | Oral allergy syndrome.mp. |
| 27. | food pollen syndrome.mp. [mp=title, book title, abstract, original title, name of substance word, subject heading word, floating sub-heading word, keyword heading word, organism supplementary concept word, protocol supplementary concept word, rare disease supplementary concept word, unique identifier, synonyms, population supplementary concept word, anatomy supplementary concept word] |
| 28. | food induced anaphylaxis/ or Food-induced anaphylaxis.mp. or anaphylaxis/ |
| 29. | milk allergy/ |
| 30. | egg allergy.mp. [mp=title, book title, abstract, original title, name of substance word, subject heading word, floating sub-heading word, keyword heading word, organism supplementary concept word, protocol supplementary concept word, rare disease supplementary concept word, unique identifier, synonyms, population supplementary concept word, anatomy supplementary concept word] |
| 31. | nut allergy/ |
| 32. | peanut allergy/ or sesame allergy/ or almond allergy/ or cashew allergy/ or apple allergy/ or egg white allergy/ or fruit allergy/ or mushroom food allergy/ or peach allergy/ or pistachio allergy/ or multiple food allergy/ or rice allergy/ or seed allergy/ or walnut allergy/ or poultry meat allergy/ or egg yolk allergy/ or hazelnut allergy/ or red meat allergy/ |
| 33. | legume allergy/ |
| 34. | fish allergy/ or shellfish allergy/ |
| 35. | Pea allergy.mp. |
| 36. | Chickpea allergy.mp. |
| 37. | Lentil allergy.mp. |
| 38. | wheat allergy/ |
| 39. | Lupin allergy.mp. |
| 40. | soy allergy/ |
| 41. | Kiwi allergy.mp. |
| 42. | Rice allergy.mp. |
| 43. | Buckwheat allergy.mp. |
| 44. | Mustard allergy.mp. |
| 45. | poppy seed allergy.mp. |
| 46. | sunflower seed allergy.mp. |
| 47. | pumpkin seed allergy.mp. |
| 48. | ("Tree nut allergy" or "Hazelnut allergy" or "Legumes allergy" or "Almond allergy" or "Cashew allergy" or "Chestnut allergy" or "Pistachio allergy" or "Walnut allergy").mp. [mp=title, book title, abstract, original title, name of substance word, subject heading word, floating sub-heading word, keyword heading word, organism supplementary concept word, protocol supplementary concept word, rare disease supplementary concept word, unique identifier, synonyms, population supplementary concept word, anatomy supplementary concept word] |
| 49. | (Sesame Allergy or Pecan allergy or Brazil nut allergy or Soy allergy or Fish allergy).mp. [mp=title, book title, abstract, original title, name of substance word, subject heading word, floating sub-heading word, keyword heading word, organism supplementary concept word, protocol supplementary concept word, rare disease supplementary concept word, unique identifier, synonyms, population supplementary concept word, anatomy supplementary concept word] |
| 50. | (Seafood allergy or Shellfish allergy).mp. [mp=title, book title, abstract, original title, name of substance word, subject heading word, floating sub-heading word, keyword heading word, organism supplementary concept word, protocol supplementary concept word, rare disease supplementary concept word, unique identifier, synonyms, population supplementary concept word, anatomy supplementary concept word] |
| 51. | (Food adj3 anaphylaxis).mp. [mp=title, book title, abstract, original title, name of substance word, subject heading word, floating sub-heading word, keyword heading word, organism supplementary concept word, protocol supplementary concept word, rare disease supplementary concept word, unique identifier, synonyms, population supplementary concept word, anatomy supplementary concept word] |
| 52. | or/21-51 |
| 53. | health care disparity/ or health disparity/ or economic inequality.mp. [mp=title, book title, abstract, original title, name of substance word, subject heading word, floating sub-heading word, keyword heading word, organism supplementary concept word, protocol supplementary concept word, rare disease supplementary concept word, unique identifier, synonyms, population supplementary concept word, anatomy supplementary concept word] |
| 54. | health inequalit*.mp. |
| 55. | health equity/ or right to health/ |
| 56. | Demography/ |
| 57. | health equality.mp. |
| 58. | differences.mp. |
| 59. | Prevalence/ |
| 60. | Public Health/ |
| 61. | or/53-60 |
| 62. | minority group/ or ethnic background/ or ethnic difference/ or ethnic group/ or racial background/ or racial disparity/ or racial diversity/ or race difference/ |
| 63. | *Ethnicity/ |
| 64. | ethnic*.mp. |
| 65. | population groups/ |
| 66. | exp ethnicity/ |
| 67. | ethnic group*.mp. |
| 68. | racial group*.mp. |
| 69. | race.ti,ab,kf. |
| 70. | sociodemographics/ |
| 71. | culture.mp. |
| 72. | cultural factor/ |
| 73. | characteristic*.mp. |
| 74. | ethnic.mp. |
| 75. | minority group*.mp. |
| 76. | African.mp. or African/ |
| 77. | black.mp. |
| 78. | Asian.mp. or Asian/ |
| 79. | Hispanic.mp. or Hispanic/ |
| 80. | Middle Easterner.mp. |
| 81. | or/62-80 |
| 82. | 20 and 52 and 61 and 81 |

**CINAHL**

(( (MH "Pediatrics") OR "pediatric* OR pediatrics* OR paediatric* or paediatrics*" ) OR ( (MH "Child+") OR "child*" OR (MH "Infant+") OR "infant" OR "baby" OR "babies" OR (MH "Child, Preschool") OR "toddler" ) OR ( (MH "Adolescence") OR "teen*" OR "adolescent" OR "adolescent*" ) OR "youth" OR "juvenile*" OR "preschool" OR "pre teen" OR "pupil*" OR (MH "Human")) AND ((MH "Food Hypersensitivity") OR (MH "Hypersensitivity, Immediate") OR (MH "Milk Hypersensitivity") OR "Immunoglobulin E" OR "IgE mediated food allergy" OR "food allerg*" OR (MH "Anaphylaxis") OR "Anaphylaxis" OR (MH "Pollen-Food Allergy") OR "Food-induced anaphylaxis" OR "egg allergy or egg hypersensitivity" OR "nut allergy" OR "peanut allergy" OR "pea allergy" OR "chickpea allergy" OR "lentil allergy" OR "wheat allergy" OR "lupin allergy" OR "soya allergy" OR "kiwi allergy" OR "rice allergy" OR "buckwheat allergy" OR "mustard allergy" OR "poppy seed allergy" OR "sunflower seed allergy" OR "pumpkin seed allergy" OR "Tree nut allergy" or "Hazelnut allergy" or "Legumes allergy" or "Almond allergy" or "Cashew allergy" or "Chestnut allergy" or "Pistachio allergy" or "Walnut allergy" OR "Sesame Allergy" or "Pecan allergy" or "Brazil nut allergy" or "Soy allergy" or "Fish allergy" OR "Seafood allergy" or "Shellfish allergy") AND ((MH "Health Inequities") OR (MH "Healthcare Disparities") OR (MH "Diversity, Equity, Inclusion") OR "health equity" OR "health inequalit*" OR "Demograph*" OR "health equality" OR (MH "Equality") Or "differences" OR (MH "Prevalence") OR (MH "Public Health Nutrition")) AND ((MH "Ethnic Groups") OR (MH "Racial Equality") OR "ethnic*" OR "ethnic group*" OR "racial group*" OR "race" OR (MH "Sociodemographic Factors") OR (MH "Culture") OR "culture" OR "characteristic*" OR (MH "Minority Groups") OR "minority group" or "African" OR "black" OR "Asian" OR "Hispanic" OR (MH "Middle Eastern Persons") OR "Middle Easterner")

**Appendix 5.** Summary of studies organized by outcome type

**Appendix 5a.** Prevalence differences by ethnicity

| **Author/ year** | **Key finding** |
| --- | --- |
| Ahmed et al., 2018 | Egg allergy: 3.3% (single non-Inuit case).  Peanut allergy: 3.3% (single Inuit case).  Tree nut allergy: 6.7% (all Inuit cases).  No sensitisation to cow’s milk, soy, or wheat.  Parent-reported food allergy: 11.4%, highest in non-Inuit (14.3%). |
| Luyt et al., 2016 | South Asian children had higher risk of allergy to almond (RR = 3.95, 95% CI 1.43–10.89, p=0.0017)  cashew (RR = 2.59; 95% CI 1.68–4.00, p=0.0001)  pistachio (RR = 3.71; 95% CI 2.23–6.16, p=0.0001).  No increased risk was found for peanut or other foods |
| Dias et al., 2008 | Non-Caucasian children made up 52.6% of the allergy clinic population versus 35.9% in general paediatric clinics (difference = 16.7%; 95% CI 5.6–27.8, p<0.01).  Non-Caucasian children had a higher average number of food allergens (2.05 vs. 1.22; mean difference = 0.83, t = 4.15, d.f. = 74, p<0.01). |
| Fox et al., 2015 | The proportion of non-White children diagnosed with peanut allergy (PA) increased significantly from 26.8% to 50.3% over 14 years (increase = 23.5%, p=0.001).  This rise was observed across most non-White subgroups, except Black-African children.  Subgroup increases: Asian Indian/Pakistani +5.99% (p = .016), Asian-Chinese +3.39% (p = .013), Mixed ethnicity +13.5% (p = .0001).  In contrast, White children showed a 23.5% decrease (p = .0001).  The increase was specific to PA, with no similar changes for egg allergy or in the hospital’s referral base. |
| Jiang et al., 2023 | Black children had the highest rate of convincing food allergy (8.9%; 95% CI 7.6–10.3) and peanut allergy (3.0%, 95% CI 2.4–3.8). They also had the highest rates of egg allergy (1.6%, 95% CI 1.0–2.7) and fin fish allergy (0.9%, 95% CI 0.6–1.5).  Asian children had the lowest rate of convincing food allergy (6.5%, 95% CI: 5.1–8.2) but reported the highest tree nut allergy (2.0%; 95% CI 1.2–3.2).  The study shows that food allergy burden disproportionately falls on non-White children and those with lower household incomes. |
| Joseph et al., 2021 | Middle Eastern/North African (MENA) patients were strongly associated with sesame allergy compared with other food allergies (OR 3.15; 95% CI 1.85–5.08) and with no food allergy (OR 2.75; 95% CI 1.63–4.41), p<0.001.  The association remained after adjustment and was stronger in allergist-confirmed cases (AOR up to 3.76, p<0.001).  Non-Hispanic Black children were less likely to have sesame allergy than other groups. |
| Joseph et al., 2016 | Sensitisation (sIgE >0.35 IU/mL) to food allergens was higher among African American children compared to non–African American children.  No significant racial/ethnic differences were observed in IgE-confirmed food allergy (IgE-FA).  A higher proportion of African American children were classified as having peanut allergy, with 1.7% exceeding the 95% predictive decision point vs 0.5% in non–African American children.  Race/ethnicity was not significantly associated with IgE-FA (AOR 1.12; 95% CI 0.58–2.17, p =0.75) but was associated with sensitisation to multiple food allergens (AOR 1.80, 95% CI 1.22–2.65, p=0.003). |
| Brewer et al., 2022 | Peanut allergy was the most common, affecting 65.3% of the cohort, with similar prevalence in Black (65.4%) and White (65.3%) children (p>0.99).  Black children had delayed introduction of peanut and milk compared to White children. White children were more likely to have early introduction (<6 months) of peanut (OR 2.6; 95% CI 1.1–7.2) and milk (OR 2.7; 95% CI 1.1–6.7), and less likely to have delayed introduction (>11 months) of peanut (OR 0.13; 95% CI 0.1–0.5) and milk (OR 0.2, 95% CI: 0.1–0.6).  Nearly 89% of Black children with peanut allergy had not been introduced to peanut by 1 year of age (or never introduced) compared to 67% of White children.  No significant racial difference was observed for egg introduction.  The study highlights significant racial disparities in peanut and milk introduction, which may contribute to higher food allergy prevalence among Black children and point to the need for targeted, culturally sensitive interventions. |
| Mahdavinia et al., 2021 | -African American children had higher odds of finfish (OR 2.54, p<0.01) and shellfish allergy (OR 3.10; p<0.001) vs White children.  -Trend towards higher odds of wheat allergy in African American children (OR 2.10; 95% CI 0.87–5.04, p=0.097).  -Trend towards lower odds of sesame allergy in African American children |

**Appendix 5b.** Access to care and management

| **Author/ year** | **Treatment** | **Key finding** |
| --- | --- | --- |
| Gallagher et al., 2025 | Oral Immune Therapy (OIT) | -White over-represented in OIT (75% vs 63.6%, p=0.0068)  -Black under-represented (4.1% vs 18%, p<0.0001)  -Black race associated with lower odds of OIT  -No significant associations in other race/ethnic groups or for neighbourhood affluence/disadvantage indices |
| Hannaway et al., 2005 | Epinephrine injector | -White students nearly 5× more likely than non-Whites to receive epinephrine (OR 4.5, p<0.001)  -Males more likely than females (OR 1.44, p<0.02) |
|  | | |
| Coleman et al., 2022 | White caregivers were more likely than Black caregivers to report access to allergen-free foods (88.1% vs 59%, p<0.001) and to purchase them online (35.2% vs 12%, p<0.001).  In unadjusted analysis, White children were 5.2 times more likely to have access than Black children (p < .001), but after adjustment, the difference was reduced 2.4 times and not statistically significant (p=0.08).  Race, socioeconomic status, and online purchasing predicted access to allergen-free foods.  Black children were more likely to live in food deserts, but food desert residence was not associated with access to allergen-free foods. | |

**Appendix 5c.** Health outcome severity

| **Author/ year** | **condition** | **Key finding** |
| --- | --- | --- |
| Buka et al., 2015 | Anaphylaxis incidence | South Asian children had a higher risk of severe anaphylaxis than White children  -South Asian children are at a greater risk of severe anaphylaxis compared to White children  OR 5.31 P=0.002  -Among the group of South Asian children, a significantly higher proportion of attendances were with severe anaphylaxis than in children who were White  [47.1% (27/51) vs 17.1% (7/41)^41^]  -The multivariate logistic regression analysis showed that the odds of South Asian children presenting with anaphylaxis were significantly higher  (OR 2.37, P=0.001) |
| Sakai-Bizmark et al., 2019 | Food-induced anaphylactic reaction | -ED visit rates for food-induced anaphylaxis were highest among Black children, with a rate of 15.26 per 100,000 children overall. This was notably higher than non-Hispanic White children (10.00) and Other Races (9.21)  -This elevated rate for Black children is primarily attributed to those living in urban regions.  -For all racial groups, the incidence of anaphylaxis-related ED visits tends to increase with older age in rural areas. Conversely, in urban regions, the highest incidence rates are observed among younger children |
| Brown et al., 2021 | Bullying, anxiety, and peer relationship | About 20% of school-aged children with food allergy experienced bullying, with no overall racial differences.  Among children ≥11 years, bullying was higher in White children than in Black children (18.2%, p =0.046).  White parents (20.7%) more often reported being teased about their child’s FA than Black parents (6.9%, p=0.004).  Black parents were more likely to intervene directly (p < .001) and more often reported their actions as beneficial (93.3% vs 81.5%, p=0.29).  No racial differences were found in composite peer relationship or overall anxiety scores, though Black respondents scored higher on specific anxiety items (“felt nervous/worried”, p=0.02).  Significant socioeconomic differences were observed: more White parents had higher education (67.9% vs 44.2%) and higher household income (≥$100,000: 74.1% vs 39.3%, p<0.001).  More Black students attended public school (76.2% vs 50.0%), while more White students attended private school (48.1% vs 14.3%, p =0.027).  White respondents were more likely to report availability of allergen-free lunch areas (48.1% vs 19.1%, p=0.006). |
| Dileep et al., 2023 | Atopic comorbidities | Black children had the highest mean Area Deprivation Index (ADI) (51.5), followed by Latin children (41.0), while White children had the lowest (24.2).  Compared with White children, Black children were significantly more likely to have asthma (OR 2.76; 95% CI 1.77–4.29) and allergic rhinitis (OR 2.50; 95% CI 1.63–3.85), even after adjusting for ADI and recruitment site. |
| Mahdavinia et al., 2022 | Gastroesophageal reflux | Black and White children with food allergy had similar rates of gastroesophageal reflux (GER), but Black children were less likely to receive treatment (43.6% untreated vs 18.1% in White children, p=0.002).  White children were more likely to be on multiple GER medications (19.2% vs 2.6%).  In White children, GER was associated with higher prevalence of milk, soy, and multiple food allergies, and lower prevalence of tree nut allergy (p< 0.05).  In Black children, GER was associated with higher prevalence of current eczema (p<0.05). |
